# Supplementary material for: Genome-wide screens identify Toxoplasma gondii determinants of parasite fitness in IFNγ-activated murine macrophages
Source: Nat Commun. 2020 Oct 16;11:5258. doi: 10.1038/s41467-020-18991-8 (PMC7567896; doi:10.1038/s41467-020-18991-8)
Supplement: Supplementary file 1 — Supplementary Information [file 41467_2020_18991_MOESM1_ESM.pdf]

Supplementary Fig. 1

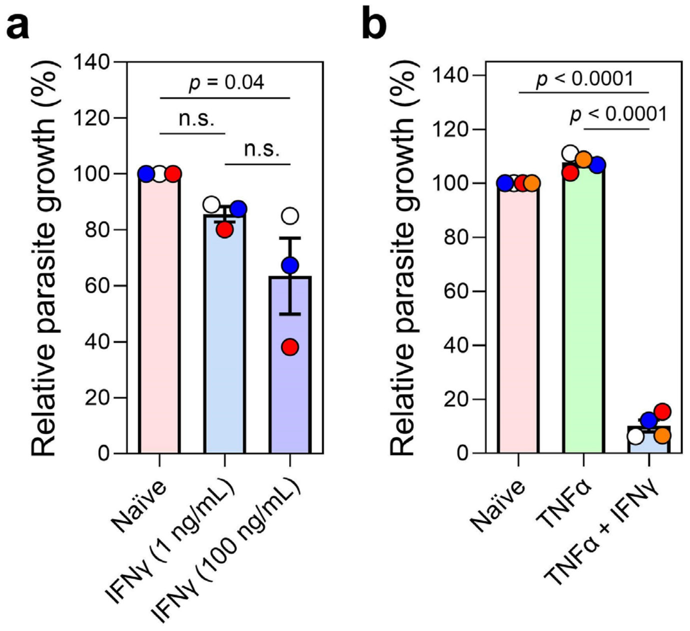

Supplementary Fig. 1 IFNγ restricts parasite growth in murine BMDMs.

(**a**) Murine BMDMs pre-stimulated with IFNγ (1 or 100 ng/mL) or left unstimulated for 24 h were infected with luciferase-expressing type I (RH) parasites for 24 h and parasite growth was measured by luciferase assay. Parasite growth in IFNγ-activated BMDMs is expressed relative to growth in naïve BMDMs. Data are displayed as mean ± SEM with independent experiments (*n* = 3) indicated by the same color dots. The significant difference was analyzed with one-way ANOVA with Tukey's multiple comparisons test.

(**b**) Murine BMDMs pre-stimulated with TNFα (10 ng/mL) or IFNγ (100 ng/mL) and TNFα (10 ng/mL) for 24 h or left unstimulated were infected with luciferase-expressing RH parasites for another 24 h and parasite growth was measured by luciferase assay. Parasite growth in stimulated BMDMs is expressed relative to growth in naïve BMDMs. Data are displayed as average ± SEM with independent experiments (*n* = 4) indicated by the same color dots. The significant difference was analyzed with one-way ANOVA with Tukey's multiple comparisons test.

Supplementary Fig. 2

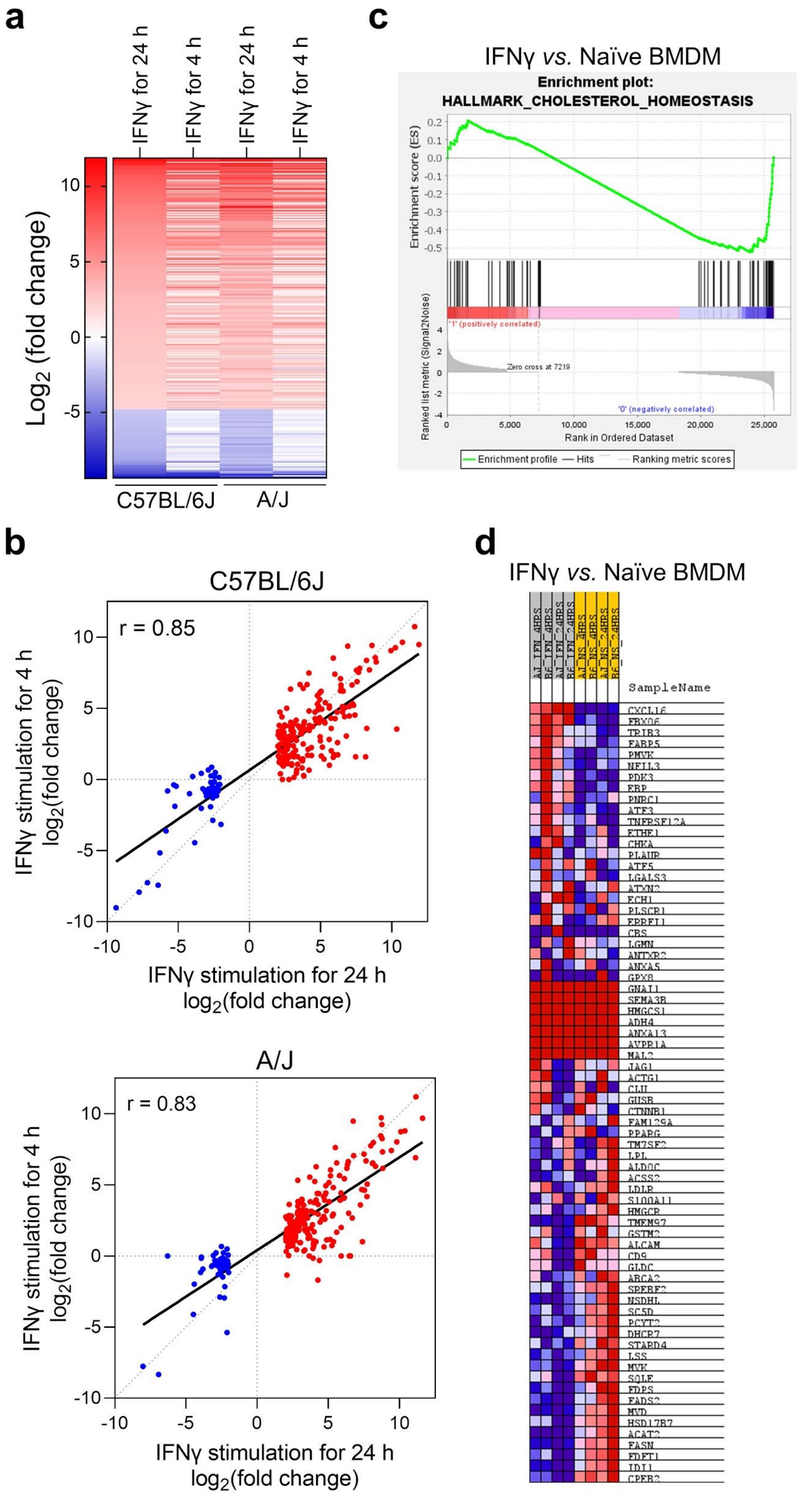

Supplementary Fig. 2 BMDM stimulation with IFNγ for 4 or 24 hours induces the expression of a similar set of genes.

(**a**) IFNγ-regulated genes in C57BL/6J and A/J BMDMs pre-stimulated with 100 ng/mL IFNγ were defined by a ≥ 4-fold change (IFNγ-activated *vs.* naïve BMDMs) in gene expression values (FPKM). Data are displayed as a heat map of $\log_2$ fold change of the 251 genes with either ≥ 4-fold upregulated (197 genes)

or ≥ 4-fold downregulated (54 genes) in IFNγ-activated BMDMs (*n* = 2). The complete set of genes is listed in Supplementary Data 3.

(**b**) Correlation between the $\log_2$ fold change of IFNγ-regulated genes after 4 and 24 h of IFNγ stimulation in C57BL/6J (left) and A/J (right) BMDMs. Perfect correlation is indicated by a grey diagonal dotted line.

(**c**) Differentially regulated pathways between IFNγ-stimulated *vs*. naïve BMDMs were identified using GSEA analysis and the MSigDB database. The 4 and 24 h time points from A/J and C57BL6/J BMDMs were treated as replicates as the goal was to identify pathways that are similarly regulated between these samples. As an example, the downregulation of the cholesterol homeostasis pathway in IFNγ-stimulated *vs*. naïve BMDMs is shown.

(**d**) An example of specific genes involved in the cholesterol homeostasis pathway (from c) regulated in IFNγ-stimulated BMDMs is presented as heat plots. Exact FPKM values of genes involved in cholesterol metabolism are in Supplementary Data 3.

Supplementary Fig. 3

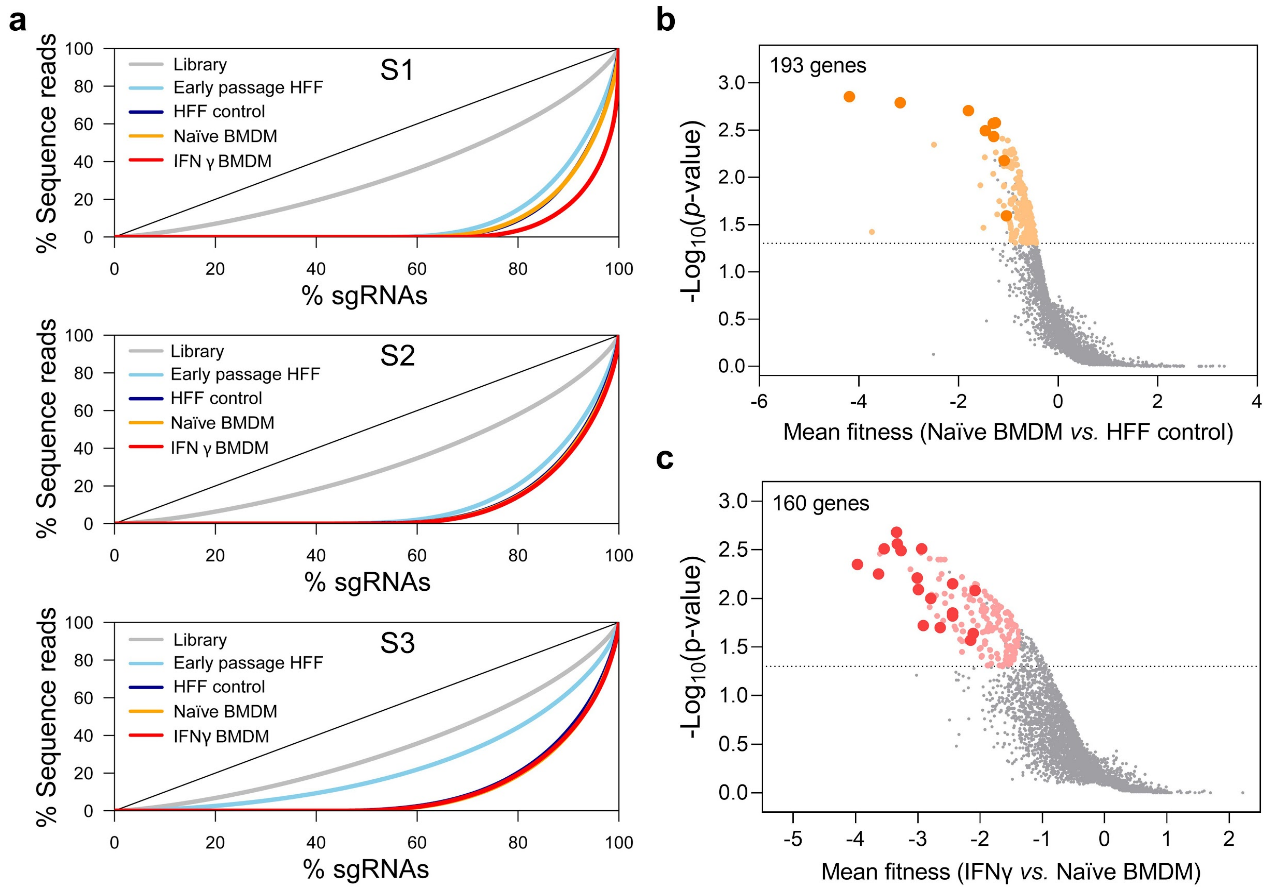

Supplementary Fig. 3 related to Fig.1.

(**a**) Potential bottlenecks were assessed by determining the abundance disparity of control guides among different samples from each screen illustrated as Lorenz curves. These curves indicate Gini coefficients for all the samples: S1, 0.33 for library, 0.80 for early passage HFF, 0.84 for HFF control, 0.84 for Naïve BMDM, and 0.90 for IFNγ BMDM; S2, 0.35 for library, 0.76 for early passage HFF, 0.80 for HFF control, 0.80 for Naïve BMDM, and 0.81 for IFNγ BMDM; S3, 0.34 for library, 0.53 for early passage HFF, 0.77 for HFF control, 0.78 for Naïve BMDM, and 0.78 for IFNγ BMDM. Some curves are overlapped due to the similarity between Gini indices.

(**b**) Scatter plot with mean fitness (Naïve BMDM *vs.* HFF control) on the x-axis and $-\log_{10}$(p-value) on y-axis. All 193 candidate genes expressed in murine macrophage[56] with $p < 0.05$ (dotted line) analyzed with One-sided Wilcoxon signed-rank test by comparing with control genes and Cohen's $d \geq 0.8$ are indicated as orange dots with dark orange dots indicating 9 high-confidence candidate genes (Table 1).

(**c**) Scatter plot with mean fitness (IFNγ *vs.* Naïve BMDM) on the x-axis and $-\log_{10}$(p-value) on y-axis. All 160 candidate genes expressed in murine macrophage[56] with $p < 0.05$ (dotted line) analyzed with One-sided Wilcoxon signed-rank test by comparing with control genes and Cohen's $d \geq 0.8$ are indicated as pink and red dots with red dots indicating 17 high-confidence candidate genes (Table 1).

Supplementary Fig. 4

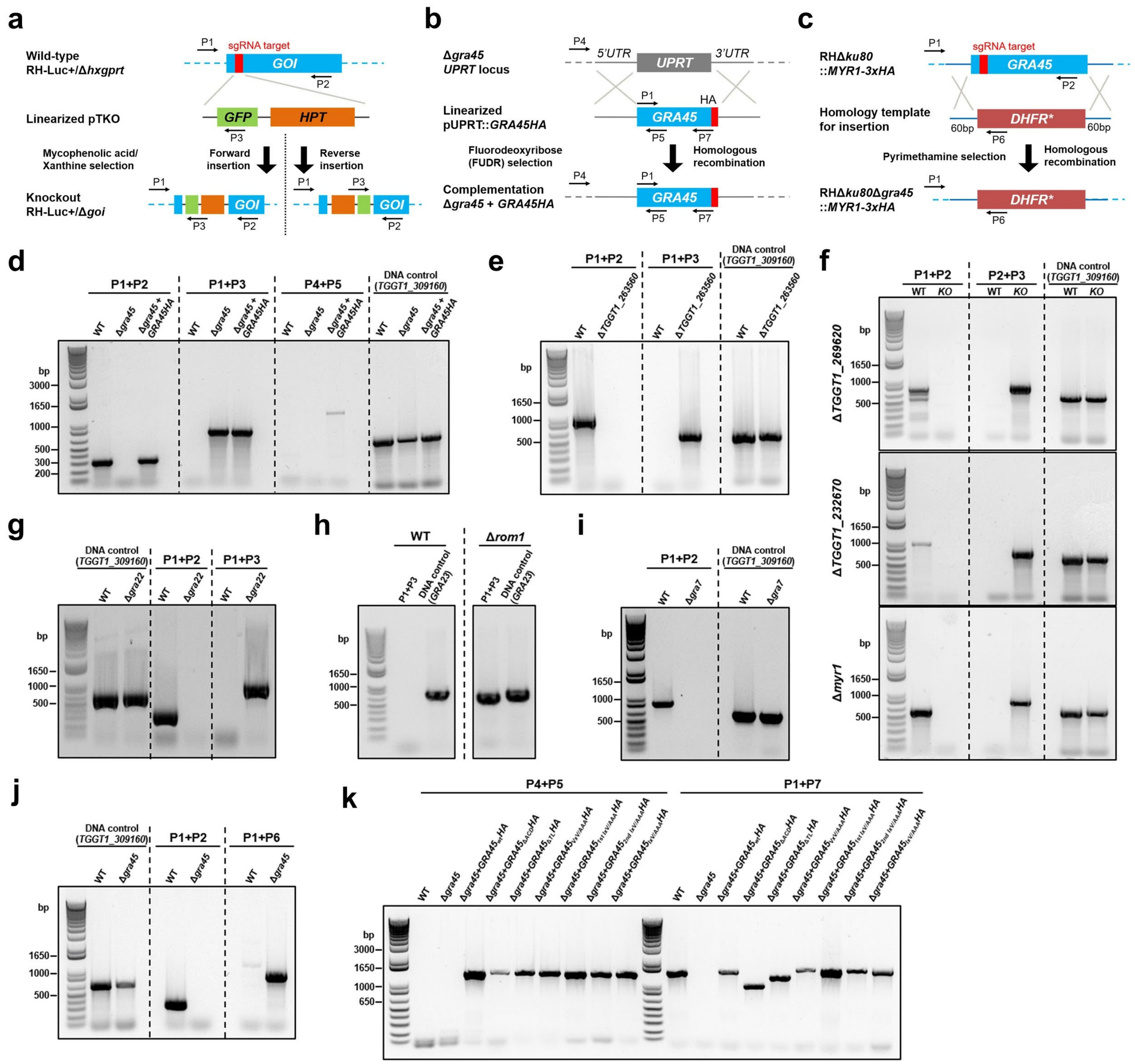

Supplementary Fig. 4 Generation and confirmation of knockout and complemented parasites.

(**a**) Schematic diagram depicting the genomic loci of the genes of interest (GOI) (top) and the CRISPR/Cas9-targeting site (red box). Linearized pTKO plasmid containing GFP-coding sequence and HXGPRT (HPT) selection cassette (middle) was used as a repair template to disrupt the GOI (bottom) after mycophenolic acid and xanthine selection. P1 and P2 refer to the primers used for confirming locus disruption. P1 + P3 or P2 + P3 are used to check insertion of the repair template into the GOI locus.

(**b**) GRA45 complementation was performed by homologous recombination of *GRA45HA*-expressing cassette (middle) into *UPRT* locus (top). The coding sequence of *UPRT* was replaced with *GRA45HA* (bottom) after FUDR selection.

(**c**) Schematic strategy used to delete the entire coding region of *GRA45* (top) by inserting DHFR* in RHΔ*ku80*::*MYR1*-*3xHA* strain. Transfection of sgRNAs targeting the *GRA45* locus (red box) together with the DHFR*-expressing amplicon flanked with 60 bp of 5-'UTR and 3'-UTR of *GRA45* (middle) after pyrimethamine selection was used to generate *GRA45* deletions.

(**d**) *GRA45* knockout in RH-Luc+/Δ*hxgprt* parasites and complementation of the gene in the *UPRT* locus.

(**e**) *TGGT1_263560* knockout in RH-Luc+/Δ*hxgprt* parasites.

(**f**) *TGGT1_269620* (top), *TGGT1_232670* (middle) and *MYR1* (bottom) knockout in RH-Luc+/Δ*hxgprt* parasites.

(**g** to **i**) Individual knockout of *GRA22* (g), *ROM1* (h) or GRA7 (i) in the RH-Cas9Δ*hxgprt* background.

(**j**) *GRA45* knockout in RHΔ*ku80*::*MYR1-3xHA* parasites.

(**k**) Complementation of mutant versions of *GRA45* in the *UPRT* locus of Δ*gra45* parasites.

All images are representative of results from 2 independent experiments.

Supplementary Fig. 5

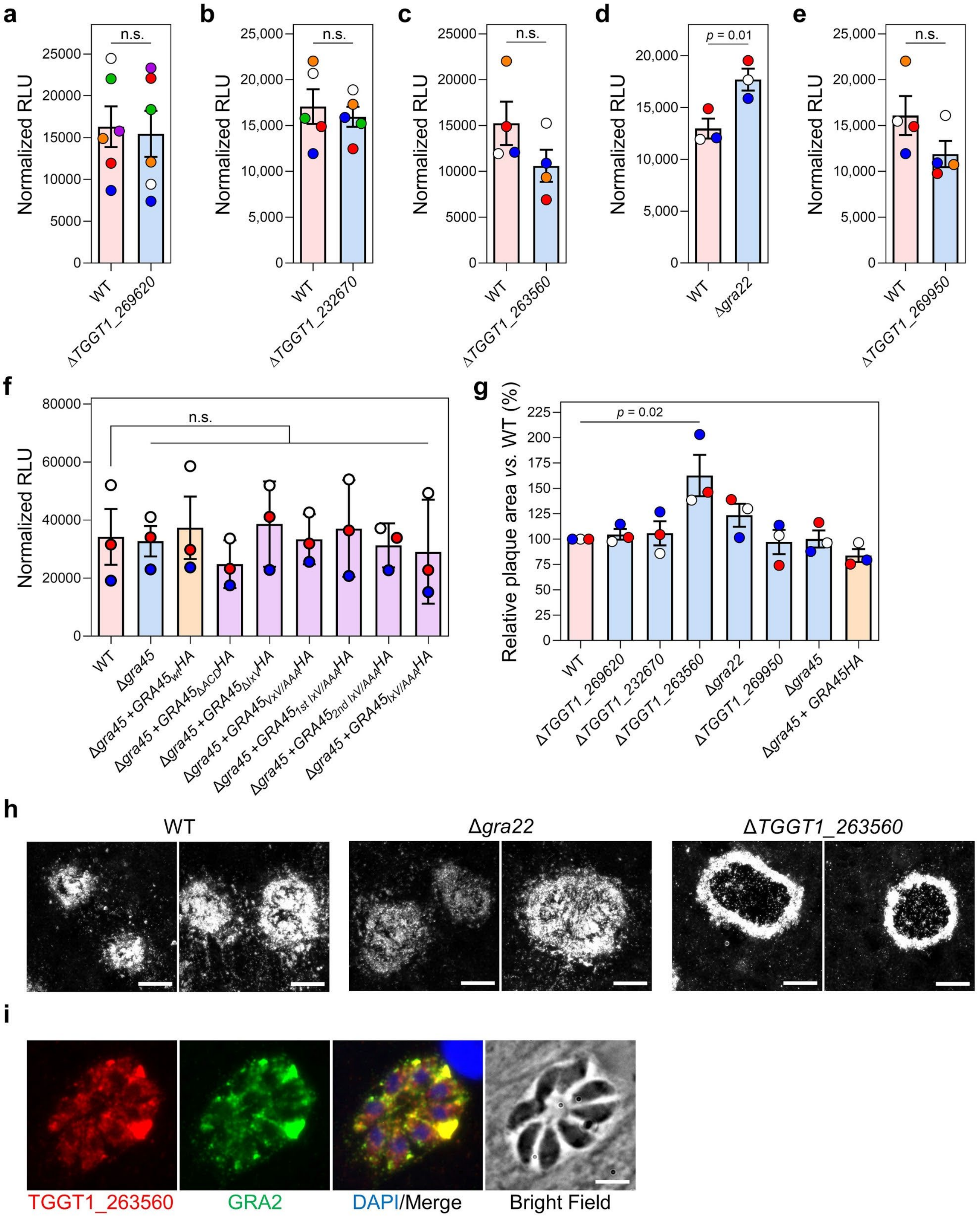

Supplementary Fig. 5 Parasite growth in naïve murine BMDMs and MEFs of individual gene knockouts identified from loss-of-function screen in IFNγ-stimulated BMDM.

(**a** to **e**) Raw luciferase reads (RLU) of WT, Δ*TGGT1_269620* (a, *n* = 6), Δ*TGGT1_232670* (b, *n* = 5), Δ*TGGT1_263560* (c, *n* = 4), Δ*gra22* (d, *n* = 3) or Δ*TGGT1_269950* (e, *n* = 4) parasites in naïve murine

BMDMs were normalized to 100% viability based on their viability measured by plaque assays. Data are displayed as mean ± SEM with independent experiments indicated by the same color dots. The significant difference between WT and knockout was analyzed with two-tailed paired *t* test.

(**f**) RLU of WT, Δ*gra45*, and Δ*gra45* complemented with C-terminal HA tagged WT or indicated mutant version of *GRA45* parasites in naïve murine BMDMs were normalized to 100% viability based on their viability measured by plaque assays. Data are displayed as mean ± SEM with independent experiments (*n* = 3) indicated by the same color dots. Not significant (n.s.), one-way ANOVA with Tukey's multiple comparisons test.

(**g**) Confluent MEFs were infected with indicated parasites for 5 days. Areas of at least 40 plaques per experiment were measured. Data are displayed as mean ± SEM with independent experiments (*n* = 3) indicated by the same color dots. The significant difference was analyzed with one-way ANOVA with Tukey's multiple comparisons test.

(**h**) Representative images of plaque size and morphology of WT, Δ*gra22*, and Δ*TGGT1_263560* parasites in MEFs (scale bar = 200 µm). The images are representative of results from at least 40 plaques examined over 3 independent experiments.

(**i**) Intracellular parasites expressing endogenously HA-tagged *TGGT1_263560* were fixed, permeabilized, and subjected to immunofluorescent assays with anti-HA (red) and anti-GRA2 (green) antibodies (scale bar = 5 µm). The images are representative of results from 2 independent experiments.

Supplementary Fig. 6

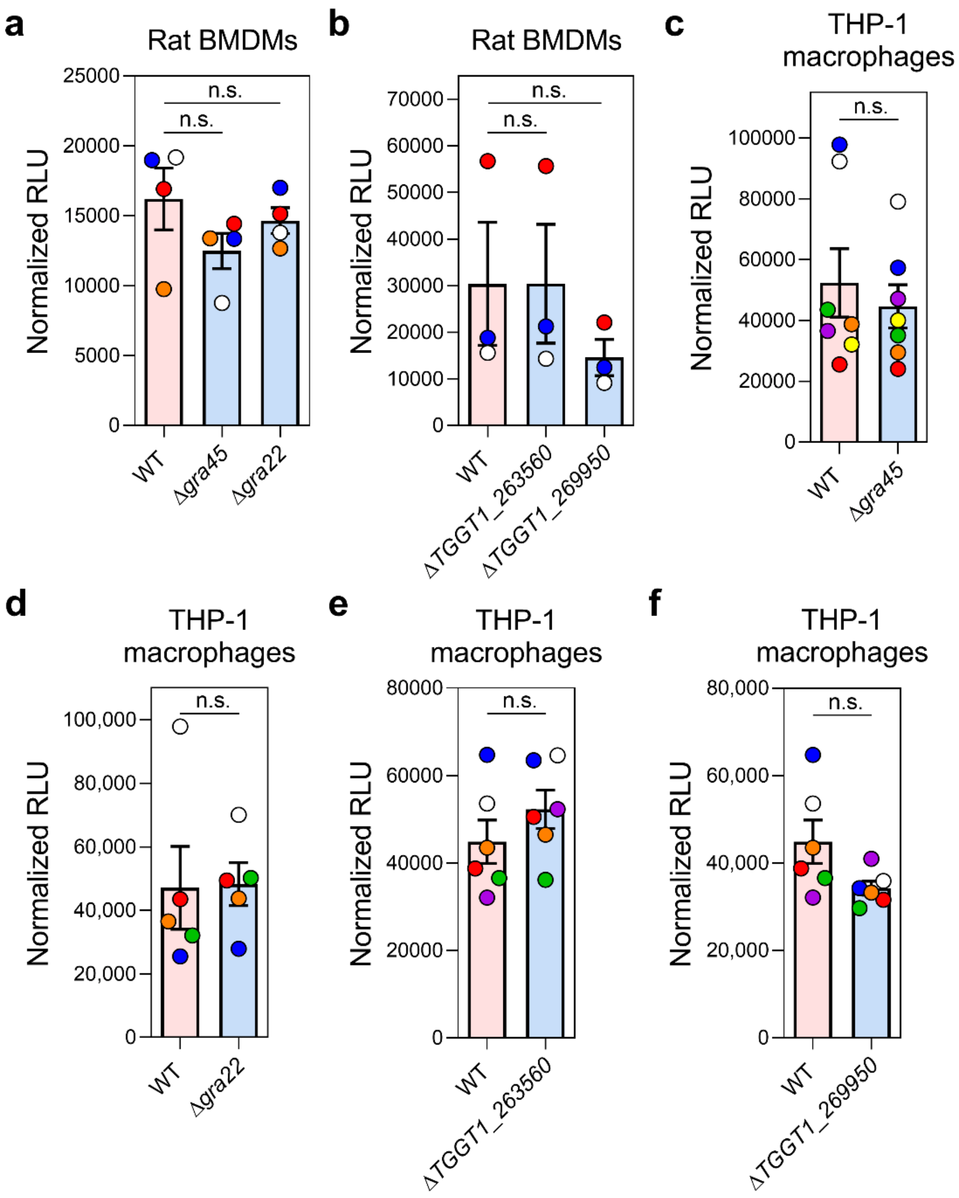

Supplementary Fig. 6 Growth of Δ*gra45*, Δ*gra22*, Δ*TGGT1_263560*, and Δ*TGGT1_269950* parasites in naïve rat BMDMs and THP-1 macrophages.

(**a** and **b**) Raw luciferase reads (RLU) of WT, Δ*gra45* (a, n = 4), Δ*gra22* (a, n = 4), Δ*TGGT1_263560* (b, n = 3) or Δ*TGGT1_269950* (b, n = 3) parasites in naïve Brown Norway rat BMDMs were normalized to 100% viability based on their viability measured by plaque assays. Data are displayed as mean ± SEM with independent experiments indicated by the same color dots. Not significant (n.s.), two-tailed paired *t* test.

(**c** to **f**) Raw luciferase reads (RLU) of WT, Δ*gra45* (c, n = 7), Δ*gra22* (d, n = 5), Δ*TGGT1_263560* (e, n = 6) or Δ*TGGT1_269950* (f, n = 6) parasites in naïve PMA-differentiated THP-1 macrophages were normalized to 100% viability based on their viability measured by plaque assays. Data are displayed as mean ± SEM with independent experiments indicated by the same color dots. Not significant (n.s.), two-tailed paired *t* test.

Supplementary Fig. 7

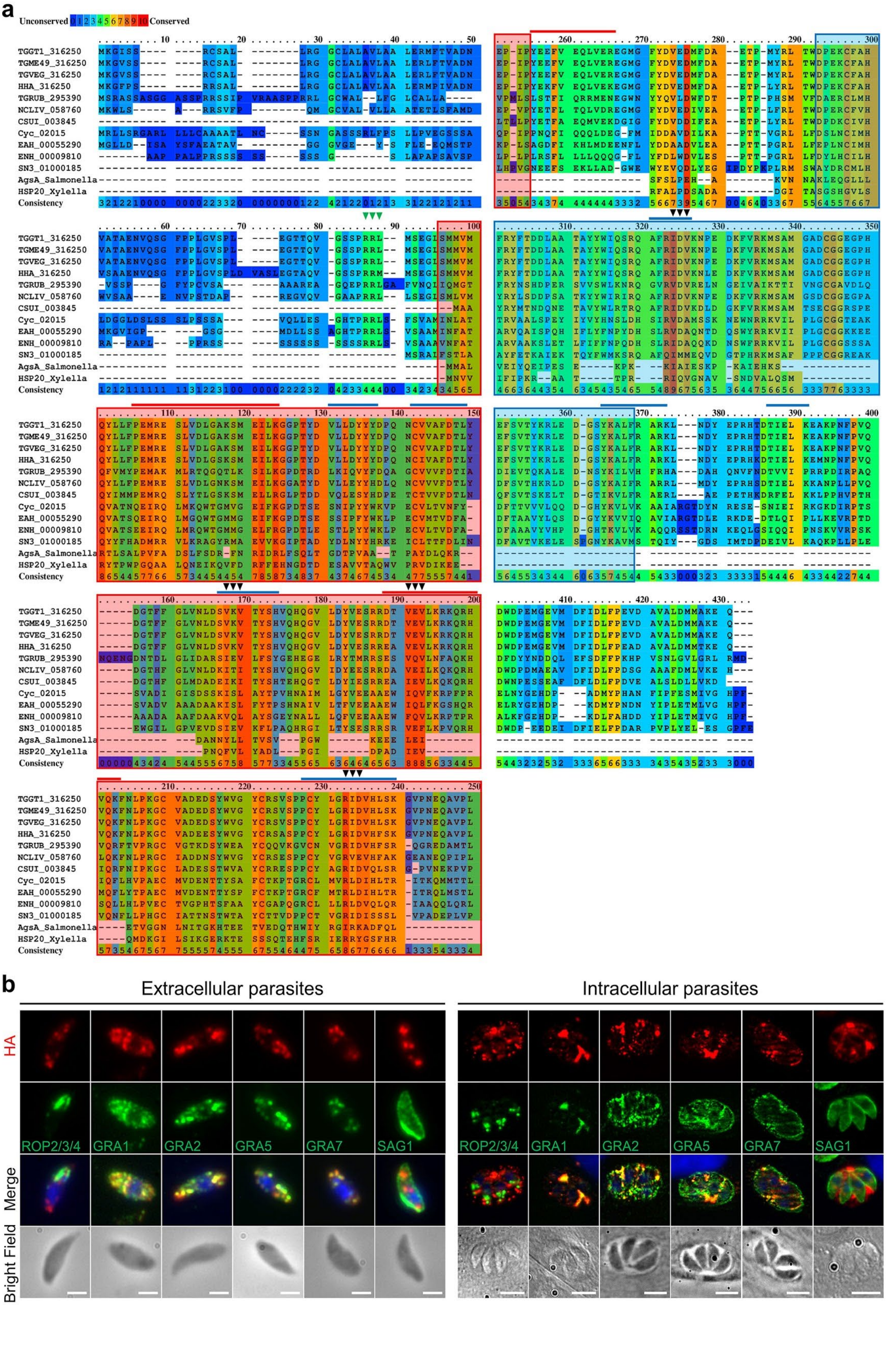

Supplementary Fig. 7 GRA45 alignment and its subcellular localization.

(**a**) Alignment of GRA45 from *Toxoplasma gondii* type I (TGGT1_316250), II (TGME49_316250) and III (TGVEG_316250); *Toxoplasma gondii* TGRUB_295390; *Hammondia hammondi* HHA_316250; *Neospora caninum* NCLIV_058760; *Cystoisospora suis* CSUI_003845; *Sarcocystis neurona* SN3_01000185; *Eimeria acervulina* EAH_00055290; *Eimeria necatrix* ENH_00009810; *Cyclospora cayetanensis* cyc_02015; HSP20 from *Xylella fastidiosa*; and AsgA from *Salmonella typhimurium*. Red boxes and blue boxes indicate predicted α-Crystallin domain (ACD) and predicted transthyretin-like fold, respectively. Predicted conserved secondary structures are indicated with red lines for helix and blue lines for strand. Green triangles and black triangles indicate the TEXEL motif and I/VxI/V motifs (VKV from amino acid 139 to 141, VEV from amino acid 162 to 164, IDV from amino acid 205 to 207, and IDV from amino acid 291 to 293), respectively. (**b**) Extracellular parasites (left panel) or intracellular parasites (right panel) expressing endogenously HA-tagged GRA45 were fixed, permeabilized, and subjected to immunofluorescent assays with the indicated antibodies. (scale bar = 2 μm for extracellular and 5 μm for intracellular parasites). The images are representative of results from 2 independent experiments.

Supplementary Fig. 8

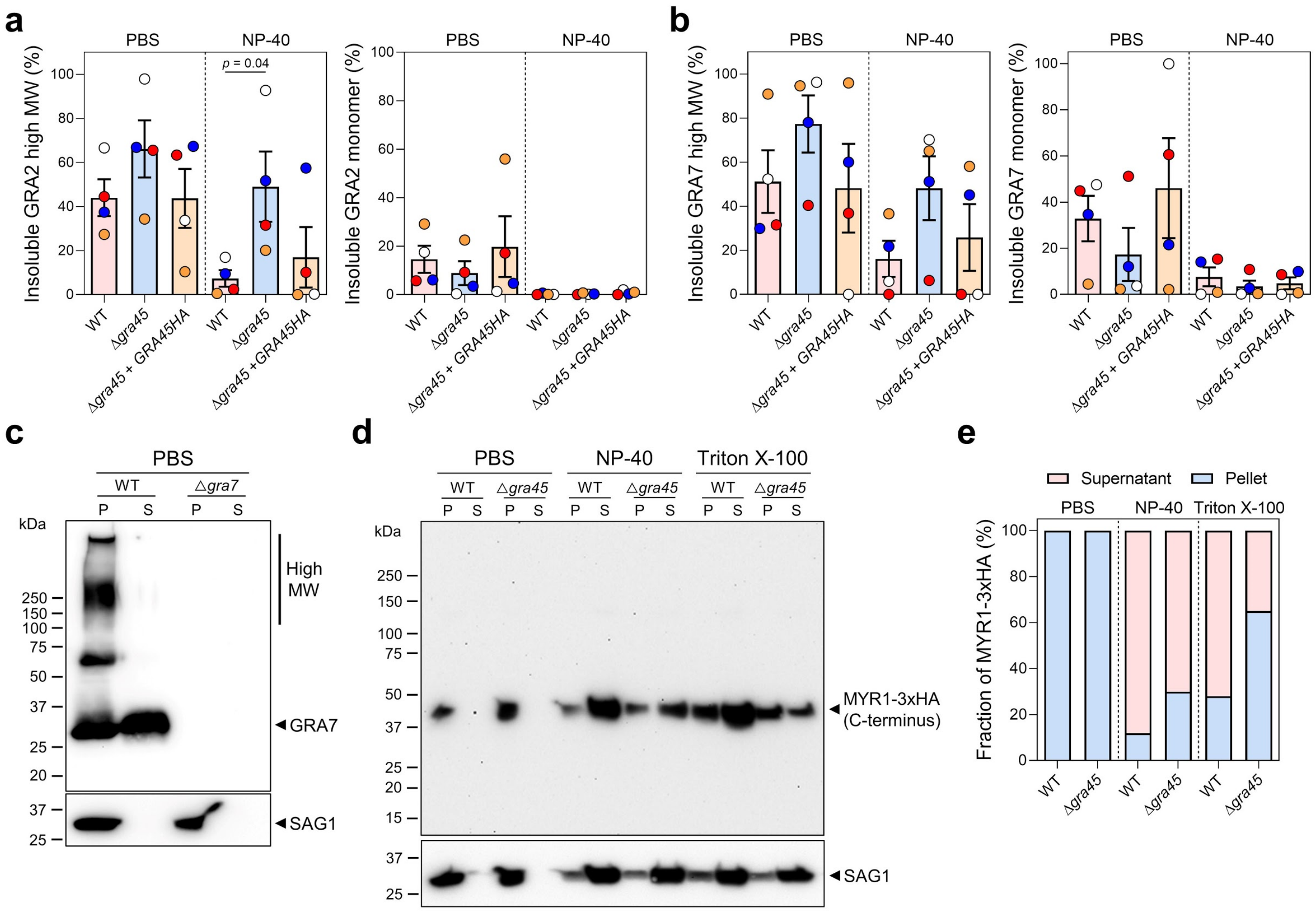

Supplementary Fig. 8

(**a**) Insoluble GRA2 was determined by the percentage of volume intensity of high molecular weight (left) or monomer (right) from the pellet fraction in both pellet and supernatant fraction from Fig. 4a. Data are displayed as mean ± SEM with independent experiments (n = 4) indicated by the same color dots. The significant difference was analyzed with two-tailed paired t test.

(**b**) Insoluble GRA7 was determined by the percentage of volume intensity of high molecular weight (left) or monomer (right) from the pellet fraction in both pellet and supernatant fraction from Fig. 4b. Data are displayed as mean ± SEM with independent experiments (n = 4) indicated by the same color dots.

(**c**) Extracellular parasites of WT or Δ*gra7* parasites were disrupted by freeze/thaw cycles followed by fractionation with high-speed centrifugation to separate the pellet (P) and supernatant (S). GRA7 were detected with anti-GRA7 antibodies. SAG1 was used as the parasite loading control. The image is result from 1 independent experiments.

(**d**) Extracellular WT parasites expressing endogenously 3xHA-tagged MYR1 or Δ*gra45* parasites in this background were disrupted by freeze/thaw cycles followed by incubating with PBS, 1% NP-40 or 1% Triton X-100 and fractionated by high-speed centrifugation to separate the pellet (P) and supernatant (S). The C-

terminal polypeptide of MYR1 was detected with anti-HA antibodies. SAG1 was used as the parasite loading control. The image is result from 1 independent experiments.

(**e**) Insoluble MYR1-3xHA was determined by the percentage of volume intensity of pellet fraction in both pellet and supernatant fraction.

Supplementary Fig. 9

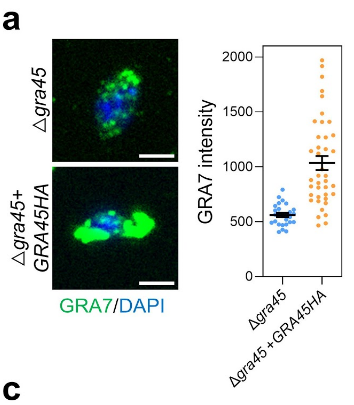

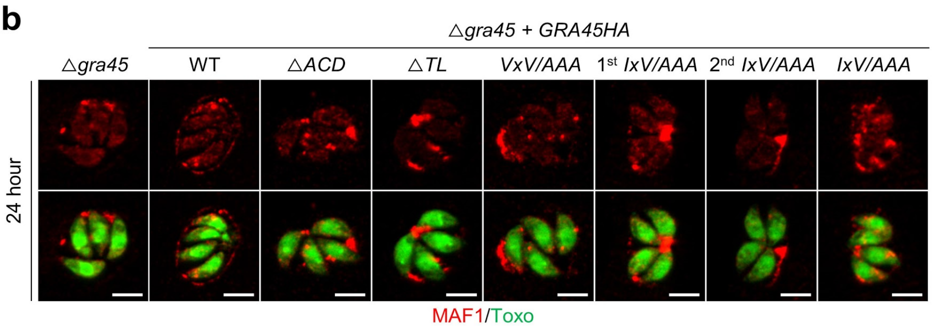

c

Parasite strain	PV lumen MAF1 (%)	
	4 h p.i.	24 h p.i.
Δgra45	99	89
Δgra45 +GRA45$_{WT}$*HA*	30	8
Δgra45 +GRA45$_{\Delta ACD}$*HA*	100	98
Δgra45 +GRA45$_{\Delta TL}$*HA*	100	100
Δgra45 +GRA45$_{VxV/AAA}$*HA*	96	89
Δgra45 +GRA45$_{1st\ IxV/AAA}$*HA*	98	87
Δgra45 +GRA45$_{2nd\ IxV/AAA}$*HA*	87	83
Δgra45 +GRA45$_{IxV/AAA}$*HA*	96	96

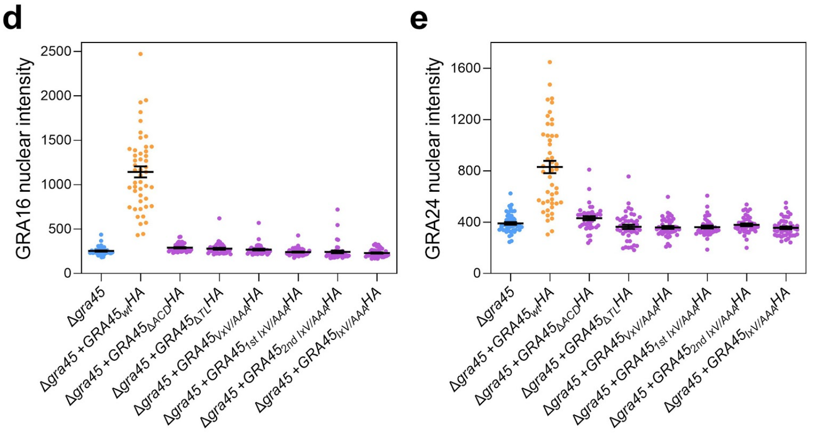

Supplementary Fig. 9 Quantification of GRA7, MAF1, GRA16 and GRA24 in Fig.5

(**a**) Extracellular Δ*gra45* or Δ*gra45* + *GRA45HA* parasites were fixed with methanol and stained with the antibodies against GRA7. On the left, the representative images are identical to Fig.5b with longer exposure (Scale bar = 2 μm). Quantification of GRA7 intensity is presented in the right panel. Data are displayed as mean ± SEM with individual dots representing single parasites (n = 27 for Δ*gra45* parasites and n = 40 for Δ*gra45* + *GRA45HA* parasites examined over 2 independent experiments).

(**b**) HFFs were infected with Δ*gra45* or Δ*gra45* complemented with wild-type or indicated mutant version of *GRA45* for 24 h followed by fixing and staining with antibodies against MAF1. The images are representative of results from 2 independent experiments and were taken at identical exposure times for each channel (scale bar = 5 μm). The images are representative of results from 2 independent experiments.

(**c**) Localization of MAF1 from Fig.5d and (b) in at least 100 vacuoles was observed and the percentage of vacuoles with only PV lumen staining was quantified and presented in the table.

(**d** and **e**) Δ*gra45* or Δ*gra45* complemented with wild-type or indicated mutant version of *GRA45* were transiently transfected with GRA16-Ty (d) or GRA24-Ty (e) expressing plasmids and immediately used to infect HFFs and fixed at 24 h p.i. and subjected to the immunofluorescent assay with antibodies against the Ty epitope. The nuclear intensity of GRA16 (d) or GRA24 (e) was quantified in host cells containing a

single PV with 4 or more parasites. Data are displayed as mean ± SEM with individual dots representing single host cell (*n* ≥ 40 cells examined over 1 independent experiment).

Supplementary Fig. 10

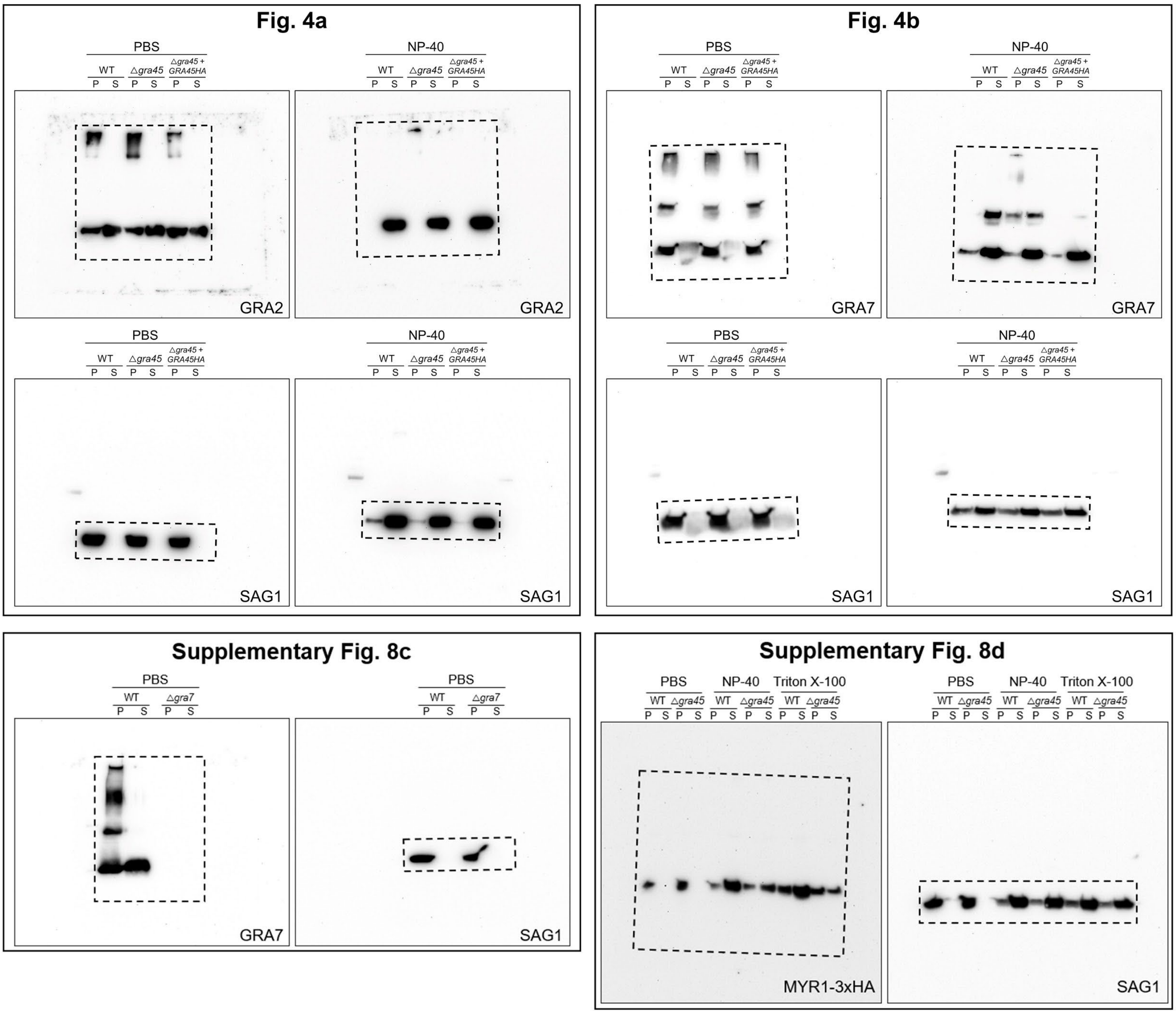

Supplementary Fig. 10 All uncropped Western blot images in this study.
