# Supplementary material for: Genome-wide screens identify Toxoplasma gondii determinants of parasite fitness in IFNγ-activated murine macrophages
Source: Nat Commun. 2020 Oct 16;11:5258. doi: 10.1038/s41467-020-18991-8 (PMC7567896; doi:10.1038/s41467-020-18991-8)
Supplement: Supplementary file 3 — Reporting Summary [file 41467_2020_18991_MOESM3_ESM.pdf]

nature research

Corresponding author(s): Jeroen P.J. Saeij

Last updated by author(s): Sep 17, 2020

Reporting Summary

Nature Research wishes to improve the reproducibility of the work that we publish. This form provides structure for consistency and transparency in reporting. For further information on Nature Research policies, see our Editorial Policies and the Editorial Policy Checklist.

Statistics

For all statistical analyses, confirm that the following items are present in the figure legend, table legend, main text, or Methods section.

n/a	Confirmed	
[ ]	[x]	The exact sample size (n) for each experimental group/condition, given as a discrete number and unit of measurement
[ ]	[x]	A statement on whether measurements were taken from distinct samples or whether the same sample was measured repeatedly
[ ]	[x]	The statistical test(s) used AND whether they are one- or two-sided<br>*Only common tests should be described solely by name; describe more complex techniques in the Methods section.*
[x]	[ ]	A description of all covariates tested
[x]	[ ]	A description of any assumptions or corrections, such as tests of normality and adjustment for multiple comparisons
[ ]	[x]	A full description of the statistical parameters including central tendency (e.g. means) or other basic estimates (e.g. regression coefficient) AND variation (e.g. standard deviation) or associated estimates of uncertainty (e.g. confidence intervals)
[ ]	[x]	For null hypothesis testing, the test statistic (e.g. F, t, r) with confidence intervals, effect sizes, degrees of freedom and P value noted<br>*Give P values as exact values whenever suitable.*
[x]	[ ]	For Bayesian analysis, information on the choice of priors and Markov chain Monte Carlo settings
[x]	[ ]	For hierarchical and complex designs, identification of the appropriate level for tests and full reporting of outcomes
[ ]	[x]	Estimates of effect sizes (e.g. Cohen's d, Pearson's r), indicating how they were calculated

Our web collection on statistics for biologists contains articles on many of the points above.

Software and code

Policy information about availability of computer code

Data collection	Illumina sequencing data were collected using NextSeq System Suite v2.2.0. sgRNA read mapping was performed using custom software as described in (Sidik et al., Nat Protoc, 2018). RNAseq data were collected using Hiseq Control Software (HCS) version 2.2.68. The RNAseq reads were mapped to the mouse genome (mm10) using Bowtie (version 2.0.2) and Tophat (version 2.0.4) and transcript abundance estimated in cufflinks (version 2.2.1).
Data analysis	CRISPR screen data were analyzed using R (www.R-project.org) version 3.6.3 and Excel (Microsoft Office) version 16.0. All statistical analyses were performed using Prism (GraphPad) version 8.0. Gene set enrichment analysis was performed using GSEA program (version 4.0.3). Protein alignments were performed using online PRALINE program (http://www.ibi.vu.nl/programs/pralinewww/).

For manuscripts utilizing custom algorithms or software that are central to the research but not yet described in published literature, software must be made available to editors and reviewers. We strongly encourage code deposition in a community repository (e.g. GitHub). See the Nature Research guidelines for submitting code & software for further information.

Data

Policy information about availability of data

All manuscripts must include a data availability statement. This statement should provide the following information, where applicable:

- Accession codes, unique identifiers, or web links for publicly available datasets
- A list of figures that have associated raw data
- A description of any restrictions on data availability

The authors declare that all data supporting the findings of this study are available within the article and its Supplementary Information files or are available from the authors upon request. CRISPR screen data including raw sequencing read counts are available in Supplementary Data 1. RNA sequencing data have been deposited in BioProject database with the accession code PRJNA664106 [http://www.ncbi.nlm.nih.gov/bioproject/664106] and are provided in Supplementary Data

3. The MSigDB are available in https://www.gsea-msigdb.org/gsea/msigdb. The source data for Figs. 2, 3, 4c-j, 5f, 6b-d, and Supplementary Figs. 1, 5a-g, 6, 8a-b, 8e, 9a, 9e-d are provided as Source Data file. Custom code used in the analysis of CRISPR screen data are available from the corresponding author (contact: Jeroen P.J. Saeij, jsaeij@ucdavis.edu) upon reasonable request.

Field-specific reporting

Please select the one below that is the best fit for your research. If you are not sure, read the appropriate sections before making your selection.

[x] Life sciences [ ] Behavioural & social sciences [ ] Ecological, evolutionary & environmental sciences

For a reference copy of the document with all sections, see nature.com/documents/nr-reporting-summary-flat.pdf

Life sciences study design

All studies must disclose on these points even when the disclosure is negative.

Sample size	Sample size determination was not performed. Sample sizes were mainly determined based on preliminary data and similar studies from other groups, and sufficient to carry out the experiments required for statistical analysis. Basically, n ≥ 3 was chosen for the experiments comparing knockout parasites to wild-type parasites, as to limit the animal number and the amount of cells used but sufficient to give a meaningful result to determine the phenotype of knockout parasites. The exact number of independent experiments was indicated in each figure legend.
Data exclusions	No data were excluded from the analyses.
Replication	All attempts at replication were successful. The number of replicates is indicated in the corresponding figure legend.
Randomization	Randomization was used for all the biological experiments. For in vivo experiments, mice were randomly assigned to groups. For experiments involving parasite in vitro infection, wells containing host cells were randomly assigned to the infection with parasites.
Blinding	For immunofluorescent assay and parasite growth assay, the investigators were blinded to group allocation during data collection and analysis. For western blot experiments, the investigators were not blinded during sample collection and processing, but all samples were handled in the same blotting conditions and exposure time. For CRISPR screen and RNA sequencing, the investigators were not blinded during sample collection and analysis, but the sequencing was performed by different researchers.

Reporting for specific materials, systems and methods

We require information from authors about some types of materials, experimental systems and methods used in many studies. Here, indicate whether each material, system or method listed is relevant to your study. If you are not sure if a list item applies to your research, read the appropriate section before selecting a response.

Materials & experimental systems

n/a	Involved in the study	
[ ]	[x]	Antibodies
[ ]	[x]	Eukaryotic cell lines
[x]	[ ]	Palaeontology and archaeology
[ ]	[x]	Animals and other organisms
[x]	[ ]	Human research participants
[x]	[ ]	Clinical data
[x]	[ ]	Dual use research of concern

Methods

n/a	Involved in the study	
[x]	[ ]	ChIP-seq
[x]	[ ]	Flow cytometry
[x]	[ ]	MRI-based neuroimaging

Antibodies

Antibodies used	Mouse monoclonal anti-GRA1 (clone TG-17.43) antibodies were purchased from Biotem (Cat#BIO.018.4). Mouse monoclonal anti-GRA2 (clone TG-17.179) and anti-GRA5 (clone TG-17.113) antibodies were purchased from BioVision (Cat#A1298 and Cat#A1299). Mouse monoclonal anti-ROP2/3/4 (clone T34A7) antibodies (described in reference 95), mouse monoclonal anti-SAG1 (clone DG52) antibodies (described in reference 96), rabbit polyclonal anti-GRA7 (described in reference 97), rabbit polyclonal anti-MAF1 (described in reference 98), and rabbit polyclonal anti-SAG1 antibodies were kindly provided by Dr. John C. Boothroyd. Rat anti-HA (3F10) antibodies were obtained from Sigma-Aldrich (Cat#11867431001). Mouse anti-Myc tag (9B11) antibodies were purchased from Cell Signaling Technology (Cat#2276S). Goat polyclonal anti-IRGA6 antibodies (Cat#sc-11090) and anti-IRGB6 antibodies (Cat#sc-11079) were purchased from Santa Cruz Biotechnology, Inc. Secondary antibodies horseradish peroxidase (HRP)-conjugated Goat anti-Mouse/Rabbit/Rat IgG were purchased from Jackson ImmunoResearch Laboratories Inc. (Cat#111-035-003/#112-035-003/ #115-035-003). Goat anti-Mouse IgG (Alexa Fluor 448/594, Cat#A11029/A11032), Goat anti-Rat IgG (Alexa Fluor 594, Cat#A11007), Goat anti-Rabbit (Alexa Fluor 488/594, Cat#A11008/A11037), Donkey anti-Rabbit IgG (Alexa Fluor 488, Cat#A21206), and Donkey anti-Goat IgG (Alexa Fluor 594, Cat#A11058) secondary antibodies were purchased from Thermo Fisher.
Validation	The antibodies obtained commercially were validated for the species and application as the instruction on the manufacturer's

website. The antibodies provided by others were validated by the providers as well as in this study.
Mouse monoclonal anti-GRA1: Species reactivity, Toxoplasma gondii; Usage, WB, ELISA, and IF.
Mouse monoclonal anti-GRA2: Species reactivity, Toxoplasma gondii; Usage, WB and IF.
Mouse monoclonal anti-GRA5: Species reactivity, Toxoplasma gondii; Usage, WB and IF.
Mouse monoclonal anti-ROP2/3/4: Species reactivity, Toxoplasma gondii; Usage, WB and IF.
Mouse monoclonal anti-SAG1: Species reactivity, Toxoplasma gondii; Usage, WB and IF.
Rabbit polyclonal anti-GRA7: Species reactivity, Toxoplasma gondii; Usage, WB and IF.
Rabbit polyclonal anti-MAF1: Species reactivity, Toxoplasma gondii; Usage, WB and IF.
Rabbit polyclonal anti-SAG1: Species reactivity, Toxoplasma gondii; Usage, WB and IF.
Rat anti-HA tag: Usage, WB, IF, dot blots, ELISA, IP, and immunocytochemistry.
Mouse anti-Myc tag: Usage, WB, IP, IF, flow cytometry, chromatin IP, and immunohistochemistry.
Goat anti-IRGA6: Species reactivity, mouse; Usage, WB , IF and ELISA.
Goat anti-IRGB6: Species reactivity, mouse and rat; Usage, WB , IF, IP and ELISA.
HRP-conjugated Goat anti-Mouse IgG: Species reactivity, mouse; Usage, WB and ELISA.
HRP-conjugated Goat anti-Rabbit IgG: Species reactivity, rabbit; Usage, WB and ELISA.
HRP-conjugated Goat anti-Rat IgG: Species reactivity, rat; Usage, WB and ELISA.
Goat anti-Mouse IgG (Alexa Fluor 448/594): Species reactivity, mouse; Usage, IF, flow cytometry, immunocytochemistry, immunohistochemistry.
Goat anti-Rat IgG (Alexa Fluor 594): Species reactivity, rat; Usage, IF, flow cytometry, and immunocytochemistry.
Goat anti-Rabbit (Alexa Fluor 488/594): Species reactivity, rabbit; Usage, IF, flow cytometry, immunocytochemistry, immunohistochemistry.
Donkey anti-Rabbit IgG (Alexa Fluor 488): Species reactivity, rabbit; Usage, IF, flow cytometry, immunocytochemistry, and immunohistochemistry.
Donkey anti-Goat IgG (Alexa Fluor 594): Species reactivity, goat; Usage, IF, flow cytometry, immunocytochemistry, and immunohistochemistry.

Eukaryotic cell lines

Policy information about cell lines

Cell line source(s)	THP-1 cells purchased from ATCC were kindly provided by Dr. Daniel A. Bachovchin. Mouse embryonic fibroblasts (MEFs) purchased from ATCC were kindly provided by Dr. Anthony Sinai. Primary human foreskin fibroblasts (HFFs) were kindly provided by Dr. John C. Boothroyd.
Authentication	None of the cell lines used were authenticated but previous RNAseq data on these lines and their morphology are consistent with their identity.
Mycoplasma contamination	All cell lines were tested negative for mycoplasma contamination.
Commonly misidentified lines (See ICLAC register)	No commonly misidentified cell lines were used.

Animals and other organisms

Policy information about studies involving animals; ARRIVE guidelines recommended for reporting animal research

Laboratory animals	Six to eight-weeks-old male/female C57BL/6J mice (Stock No: 000664) and A/J mice (Stock No: 000646) were purchased from The Jackson Laboratory. Six to eight-weeks-old CD-1 female mice (Strain Code: 022) were purchased from Charles River Laboratories. Six-weeks-old old female Brown Norway rats (Strain Code: 091) were purchased from Charles River Laboratories. Mice and rats were housed in ventilated cages on corn bedding and provided with water and chow ad libitum. Cages were all on one rack at a housing density of five mice per cage and three rats per cage. The mice and rats were allowed to acclimatize in our vivarium for at least a week undisturbed. The animal room was on a 12 light/12 dark cycle, the temperature was maintained at 22–25°C, and the humidity range was 30% to 70%. Mice were monitored twice daily by veterinarians, weighed daily, and cage bedding changed every two weeks. Mice and rats were housed under pathogen-specific free conditions at the University of California, Davis animal facility.
Wild animals	The study did not involve wild animals.
Field-collected samples	The study did not involve samples collected from the field.
Ethics oversight	All animal experiments were performed in strict accordance with the recommendations in the Guide for the Care and Use of Laboratory Animals of the National Institutes of Health and the Animal Welfare Act, approved by the Institutional Animal Care and Use Committee at the University of California, Davis (UC Davis) (assurance number A-3433-01).

Note that full information on the approval of the study protocol must also be provided in the manuscript.
