# Supplementary material for: Genome-wide screens identify Toxoplasma gondii determinants of parasite fitness in IFNγ-activated murine macrophages
Source: Nat Commun. 2020 Oct 16;11:5258. doi: 10.1038/s41467-020-18991-8 (PMC7567896; doi:10.1038/s41467-020-18991-8)
Supplement: Supplementary file 4 — Description of Additional Supplementary Files [file 41467_2020_18991_MOESM4_ESM.pdf]

Description of Additional Supplementary Files

File Name: Supplementary Data 1
Description: This table contains the raw sgRNA reads for each experiment, the fitness scores, and a list with all Toxoplasma genes and all associated screen information.

File Name: Supplementary Data 2
Description: Gene ontology (GO) analysis (ToxoDB.org) was performed on the 193 or 160 candidate Toxoplasma genes determining the fitness in naïve murine BMDMs or IFNγ-activated BMDMs (see Supplementary Data 1).

File Name: Supplementary Data 3
Description: Sheet 1 “FPKM”: RNAseq data of A/J and C57BL/6J BMDMs stimulated with IFNγ for 4 or 24 h or left unstimulated. FPKM values are indicated. Genes involved in the cholesterol synthesis pathway are indicated as are pathways mediating cholesterol biosynthesis. Sheet 2 “GSEA_IFN4-24 vs NS4-24”: Gene Set Enrichment Analysis (GSEA) was performed on the BMDMs stimulated with IFNγ vs. non-stimulated (NS) BMDMs. For this analysis the A/J, C57BL/6J at 4 and 24 h were treated as four replicates to identify pathways regulated regardless of mouse strain or duration of stimulation. All data in sheet 1 was used and genes were ranked based on the differential expression between the two conditions identified using the Signal2Noise option in the stand-alone version of GSEA. KEGG and Hallmark gene sets from the MSigDB database were used to identify positively or negatively enriched gene sets. A p-value cut-off of 0.05 was used but FDRs are also displayed. 1,000 gene set permutations were used to determine p-values and FDRs.

File Name: Supplementary Data 4
Description: Primers and oligos used in this study.
